# Diagnostic and Prognostic Value of Aminoterminal Prohormone of Brain Natriuretic Peptide in Heart Failure with Mildly Reduced Ejection Fraction Stratified by the Degree of Renal Dysfunction

**DOI:** 10.3390/jcm13020489

**Published:** 2024-01-16

**Authors:** Tobias Schupp, Mohammad Abumayyaleh, Kathrin Weidner, Felix Lau, Alexander Schmitt, Marielen Reinhardt, Noah Abel, Jan Forner, Muharrem Akin, Mohamed Ayoub, Kambis Mashayekhi, Thomas Bertsch, Ibrahim Akin, Michael Behnes

**Affiliations:** 1 Department of Cardiology, Angiology, Haemostaseology and Medical Intensive Care, University Medical Centre Mannheim, Medical Faculty Mannheim, Heidelberg University, 68167 Mannheim, Germany; 2 Department of Cardiology, St. Josef-Hospital, Ruhr-Universität Bochum, 44791 Bochum, Germany; 3 Division of Cardiology and Angiology, Heart Center University of Bochum, 32545 Bad Oeynhausen, Germany; 4 Department of Internal Medicine and Cardiology, Mediclin Heart Centre Lahr, 77933 Lahr, Germany; 5 Institute of Clinical Chemistry, Laboratory Medicine and Transfusion Medicine, Nuremberg General Hospital, Paracelsus Medical University, 90419 Nuremberg, Germany

**Keywords:** heart failure with mildly reduced ejection fraction, HFmrEF, NT-proBNP, acute decompensated heart failure, biomarkers, mortality

## Abstract

Limited data concerning the diagnostic and prognostic value of blood-derived biomarkers in heart failure with mildly reduced ejection fraction (HFmrEF) is available. This study investigates the diagnostic and prognostic value of aminoterminal prohormone of brain natriuretic peptide (NT-proBNP) in patients with HFmrEF, stratified by the estimated glomerular filtration rate (eGFR). Consecutive patients with HFmrEF were retrospectively included at one institution from 2016 to 2022. First, the diagnostic value of NT-proBNP for acute decompensated heart failure (ADHF) was tested. Thereafter, the prognostic value of NT-proBNP levels was tested for 30-months all-cause mortality in patients with ADHF. From a total of 755 patients hospitalized with HFmrEF, the rate of ADHF was 42%. Patients with ADHF revealed higher NT-proBNP levels compared to patients without (median 5394 pg/mL vs. 1655 pg/mL; *p* = 0.001). NT-proBNP was able to discriminate ADHF with an area under the curve (AUC) of 0.777 (*p* = 0.001), with the highest AUC in patients with eGFR ≥ 60 mL/min (AUC = 0.800; *p* = 0.001), and no diagnostic value was seen in eGFR < 30 mL/min (AUC = 0.576; *p* = 0.210). Patients with NT-proBNP levels > 3946 pg/mL were associated with higher rates of all-cause mortality at 30 months (57.7% vs. 34.4%; HR = 2.036; 95% CI 1.423–2.912; *p* = 0.001), even after multivariable adjustment (HR = 1.712; 95% CI 1.166–2.512; *p* = 0.006). In conclusion, increasing NT-proBNP levels predicted the risk of ADHF and all-cause mortality in patients with HFmrEF and preserved renal function; however, NT-proBNP levels were not predictive in patients with HFmrEF and eGFR < 30 mL/min.

## 1. Introduction

Although improvements in the management of coronary artery disease (CAD) and heart failure (HF) stabilized the incidence of HF over the past years, HF still affects about 64 million people worldwide, with a corresponding prevalence of 4% in the general population [1,2,3]. Related to an increasing number of individuals with cardiac and non-cardiac comorbidities [4,5], related to the higher supply of invasive cardiac devices, higher rates of coronary revascularization, and cardiac pharmacotherapies, risk stratification for HF patients has even become more difficult and complex [6,7,8,9]. Even multi-morbid patients have the highest risk of acute decompensated heart failure (ADHF), which, by now, represents one of the leading causes of hospitalization in the Western world [10]. ADHF is characterized by an increased risk of cardiovascular mortality in patients with HF with reduced (i.e., HFrEF) and preserved (i.e., HfpEF) left ventricular ejection fraction (LVEF) [10]. Recently, our study group demonstrated adverse long-term prognosis in patients with ADHF and HF with mildly reduced ejection fraction (HFmrEF) compared to patients without ADHF. The rate of ADHF was 22% in patients with HFmrEF [11].

By now, many biomarkers have been evaluated to identify individuals with ADHF; whereas, specifically, the measurement of brain natriuretic peptide (BNP) and aminoterminal prohormone of brain natriuretic peptide (NT-proBNP) was embedded into daily clinical practice [12,13,14,15,16,17]. Although the predictive value of NT-proBNP levels may differ across the spectrum of HF stratified by LVEF, Savarese et al. recently demonstrated the discriminative capacity of NT-proBNP in patients with HFmrEF [18]. A decline in NT-proBNP levels was associated with improved mortality rates [19]. Contrarily, other studies concluded a limited prognostic impact of NT-proBNP values in patients with HFmrEF [20]. In patients with HF, levels of NT-proBNP may further reflect patients’ comorbidities; as such, a strong inverse correlation between renal function and NT-proBNP levels was demonstrated [20,21,22,23]. Concerning the discriminatory capacity of NT-proBNP, the prognostic value of NT-proBNP levels was more pronounced in patients with preserved renal function among patients undergoing cardiac surgery; whereas, the prognostic value of NT-proBNP levels was poor in patients with impaired renal function [24]. However, the diagnostic and prognostic value of NT-proBNP in patients with HFmrEF, stratified by renal function has never been investigated.

The present study sought to investigate (1) the diagnostic value of NT-proBNP levels to discriminate ADHF in patients with HFmrEF, as well as (2) the prognostic value of NT-proBNP levels in patients with ADHF, stratified by the presence and severity of concomitant renal dysfunction.

## 2. Materials and Methods

### 2.1. Study Patients, Design, and Data Collection

The aim of the present study was to evaluate the diagnostic capacity of NT-proBNP for ADHF. Additionally it was aimed to investigate the prognostic value of NT-proBNP for long-term all-cause mortality. All consecutive patients hospitalized with HFmrEF at one university medical centre were included from January 2016 to December 2022, as recently published [11]. Using the electronic hospital information system, all relevant clinical data related to the index event were documented, such as baseline characteristics; vital signs on admission; prior medical history; prior medical treatment; length of index hospital and intensive care unit (ICU) stay; laboratory values; data derived from all non-invasive or invasive cardiac diagnostics and device therapies, such as echocardiographic data; coronary angiography and data being derived from prior or newly implanted cardiac devices. Every re-visit at the outpatient clinic or rehospitalization related to HF or adverse cardiac events was documented until the end of the year 2022.

The present study is derived from the “Heart Failure With Mildly Reduced Ejection Fraction Registry” (HARMER), representing a retrospective single-center registry including consecutive patients with HFmrEF hospitalized at the University Medical Centre Mannheim (UMM), Germany (clinicaltrials.gov identifier: NCT05603390). The registry was carried out according to the principles of the Declaration of Helsinki and was approved by the Medical Ethics Committee II of the Medical Faculty Mannheim, University of Heidelberg, Germany (ethical approval code: 2022-818). No written informed consent was deemed necessary for the present study.

### 2.2. Inclusion and Exclusion Criteria

All consecutive patients ≥18 years of age hospitalized with HFmrEF at one institution were included, irrespective of the department of hospital admission. The diagnosis of HFmrEF was determined according to the “2021 ESC Guidelines for the diagnosis and treatment of acute and chronic heart failure” [25]. Accordingly, all patients with a LVEF of 41–49% and symptoms and/or signs of HF were included. The presence of elevated NT-proBNP levels and the evidence of structural heart disease were considered to make the diagnosis more likely but were not mandatory for diagnosis of HFmrEF. Transthoracic echocardiography was performed by cardiologists during routine clinical practice in accordance with current European guidelines [26,27]. Echocardiographic operators were blinded to the final study analyses. Patients without measurements of NT-proBNP levels and/or estimated glomerular filtration rate (eGFR) during index hospitalization were excluded. No further exclusion criteria were applied.

### 2.3. Measurement of Creatinine, eGFR, and NT-proBNP Levels

Measurements of creatinine were carried out predominantly using lithium heparinate plasma. This assay is a modification of the Jaffé method with blank correction and axis segment adjustment. This blank correction is used to minimize interferences with bilirubin. The assay was carried out on a clinical chemistry analyzer (Atellica CH 930, Siemens Healthineers, Erlangen, Germany). A linear measurement range in plasma of 0.15 mg/dL (13 μmol/L) to 30.00 mg/dL (2652 μmol/L) was expandable to 60 mg/dL (5304 μmol/L) by automated dilution. The manufacturer specifies a limit of detection (LoD) of ≤0.10 mg/dL (≤9 μmol/L) and a limit of quantitation (LoQ) of ≤0.30 mg/dL. The reference range for healthy is given as 0.55–1.02 mg/dL (49–90 μmol/L) for women and 0.70–1.30 mg/dL (62–115 μmol/L) for men. Additionally, eGFR was estimated by using the CKD-EPI formula. This is more accurate compared to the MDRD formula in estimating the eGFR in the threshold region of beginning renal insufficiency [28,29].

Measurements of NT-proBNP were performed as a direct chemiluminescence sandwich immunoassay on the Atellica Solution IM (Siemens Healthineers, Erlangen Germany). The linear quantification range of the assay for serum and plasma is 35–35,000 pg/mL (4.13–4130 pmol/L). The clinical decision threshold for the NT-proBNP assay to separate healthy from sick patients is 125 pg/mL for patients aged < 75 years and 450 pg/mL for patients aged ≥75 years.

### 2.4. Study Endpoints

First, the diagnostic value of NT-proBNP for the diagnosis of ADHF was tested within the entire study cohort. ADHF was defined according to current European guidelines [25] based on congestion, characterized by the apparent worsening of clinical signs and/or symptoms of HF requiring intravenous diuretic therapy.

The prognostic impact of NT-proBNP levels was tested for long-term all-cause mortality at 30 months in patients with ADHF at index hospitalization. All-cause mortality was documented using the electronic hospital information system and by directly contacting state resident registration offices (‘Bureau of Mortality Statistics’).

### 2.5. Statistical Methods

Quantitative data was presented as the mean ± standard error of the mean (SEM), median and interquartile range (IQR), and ranges depending on the distribution of the data. They were compared using the Student’s *t*-test for normally distributed data or the Mann–Whitney U test for non-parametric data. Deviations from a Gaussian distribution were tested by the Kolmogorov–Smirnov test. Qualitative data was presented as absolute and relative frequencies and were compared using the Chi-square test or the Fisher’s exact test, as appropriate.

C-statistics were applied by calculating the receiver operating characteristic (ROC) curves and investigating the corresponding areas under the curves (AUCs) to assess (1) the diagnostic performance of NT-proBNP levels with regard to the diagnosis of ADHF during index hospitalization, as well as (2) the prognostic performance of NT-proBNP with regard to 30-month all-cause mortality in patients with ADHF. ROC analyses were performed within the entire study cohort, as well as stratification by eGFR, including patients with eGFR ≥ 60 mL/min, ≥30–<60 mL/min, and <30 mL/min. Optimal cut-offs were determined in accordance with the maximum Youden index. AUCs for the diagnostic and prognostic performance stratified by eGFR were compared by the method of Hanley et al. [30]. Thereafter, Kaplan–Meier analyses were performed according to NT-proBNP levels based on the optimal cut-off and univariable hazard ratios (HRs) were given together with 95% confidence intervals. The prognostic impact of NT-proBNP levels was finally investigated within multivariable Cox regression models.

The results of all statistical tests were considered significant at *p* ≤ 0.05. SPSS (Version 28, IBM, Armonk, NY, USA) was used for statistics.

## 3. Results

### 3.1. Study Population

**Figure 1:**
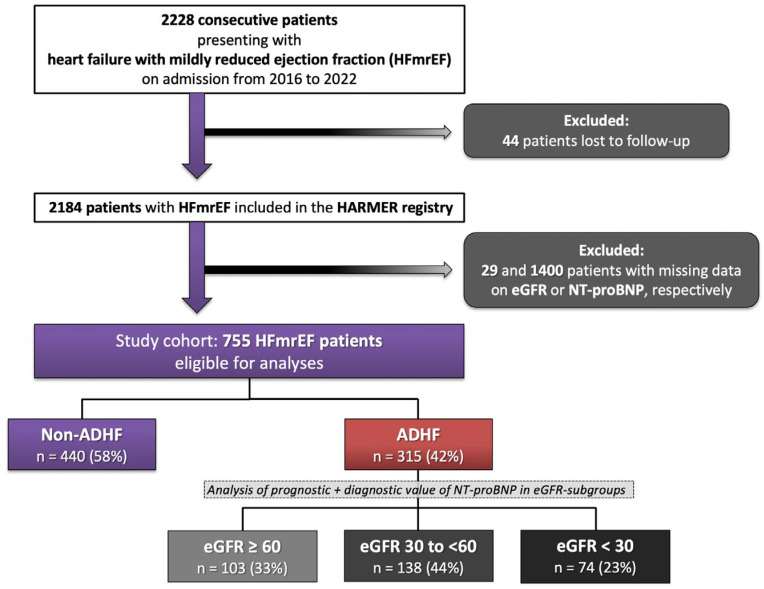
Flow chart of the study population.

A total of 2228 consecutive patients were hospitalized with HFmrEF from 2016 to 2022. In addition, 44 patients with incomplete follow-up, 1400 patients without measurements of NT-proBNP, and 29 patients without measurement of eGFR during index hospitalization were excluded. The final study cohort comprised 755 patients with HFmrEF (Figure 1; Flow chart).

Of those, 315 patients (42%) presented with ADHF. Compared to patients without ADHF, patients with ADHF were older (median age 80 years vs. 70 years; *p* = 0.001) and more commonly females (42.5% vs. 30.9%; *p* = 0.001) (Table 1). Furthermore, the rates of prior congestive HF (49.2% vs. 30.7%; *p* = 0.001) and the proportion of patients hospitalized with ADHF with <12 months to index hospitalization (20.0% vs. 10%; *p* = 0.001) were higher in patients with ADHF. In line with this, patients with ADHF had higher rates of chronic kidney disease (53.3% vs. 22.0%; *p* = 0.001), arterial hypertension (85.4% vs. 71.8%; *p* = 0.001), and diabetes mellitus (49.2% vs. 30.9%; *p* = 0.002). In contrast, the rates of ST-segment elevation AMI (STEMI) (15.9% vs. 4.4%; *p* = 0.001) and non-ST-segment elevation AMI (NSTEMI) (16.8% vs. 11.1%; *p* = 0.028) were higher in the non-ADHF group. With regard to HF etiology, ischemic cardiomyopathy was the most common cause of HF in both groups, with higher rates in patients without ADHF (65.2% vs. 56.5%; *p* = 0.001) (Table 2). In line with this, the rates of moderate to severe aortic stenosis (13.3% vs. 5.9%; *p* = 0.001), as well as aortic (8.9% vs. 3.4%; *p* = 0.001), mitral (27.3% vs. 8.2%; *p* = 0.001) and tricuspid regurgitation (33.3% vs. 11.1%; *p* = 0.001), were higher in patients with ADHF. With regard to laboratory data, patients with ADHF presented with higher creatinine (median 1.35 mg/dL vs. 1.01 mg/dL; *p* = 0.001), higher C-reactive protein (median 21 mg/L vs. 14 mg/L; *p* = 0.001), and lower hemoglobin levels (median 11.2 g/dL vs. 12.8 g/dL; *p* = 0.001) compared to patients without. Finally, patients with ADHF had higher rates of aldosterone antagonists (24.4% vs. 14.4%; *p* = 0.001) and loop diuretics (90.5% vs. 39.2%; *p* = 0.001) at discharge.

### 3.2. Correlations of NT-proBNP with Clinical, Echocardiographic, and Laboratory Data

In patients hospitalized with HFmrEF, NT-proBNP levels on admission correlated with age and body mass index, as well as with echocardiographic data, such as LVEF and TAPSE (Table 3). Among other laboratory values, inverse correlations with eGFR (r = −0.476; *p* = 0.001) and hemoglobin (r = −0.466; *p* = 0.001) were observed.

### 3.3. Diagnostic Value of NT-proBNP Levels Regarding the Presence of ADHF Stratified by eGFR

**Figure 2:**
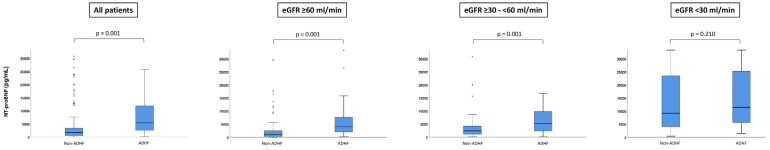
Box plots demonstrating the distribution of NT-pro BNP levels among patients with HFmrEF stratified by patients with ADHF vs. without ADHF. Data are presented as the median with interquartile ranges (boxes) and 5–95% percentiles (whiskers).

Compared to patients without ADHF, NT-proBNP levels were higher in those with ADHF within the entire study cohort (median 5394 pg/mL vs. 1655 pg/mL; *p* = 0.001), as well as in patients with eGFR ≥ 60 mL/min (median 3940 pg/mL vs. 1100 pg/mL; *p* = 0.001) and eGFR ≥ 30–<60 mL/min (median 5155 pg/mL vs. 2388 pg/mL; *p* = 0.001); whereas, NT-proBNP levels did not differ in patients with or without ADHF in the sub-group of eGFR < 30 mL/min (median 11,443 pg/mL vs. 9169 pg/mL; *p* = 0.210) (Figure 2).

**Figure 3:**
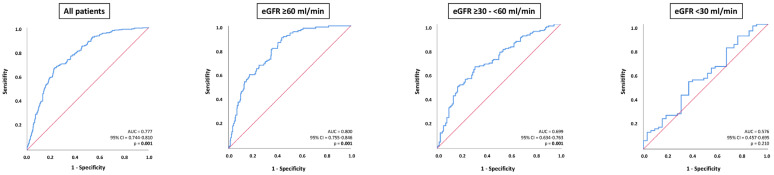
Receiver operator characteristic (ROC) curve analyses for the discrimination of ADHF from non-ADHF within the entire study cohort, as well as stratification by patients with eGFR ≥ 60 mL/min, eGFR ≥ 30–<60, and eGFR.

NT-pro BNP levels were able to discriminate patients with ADHF from those without within the entire study cohort (AUC = 0.777; 95% CI 0.744–0.810; *p* = 0.001), as well as in patients with eGFR ≥ 60 mL/min (AUC = 0.800; 95% CI 0.755–0.846; *p* = 0.001) and in patients with eGFR ≥ 30–<60 mL/min (AUC = 0.699; 95% CI 0.634–0.763; *p* = 0.001). In contrast, NT-proBNP levels were not associated with ADHF in patients with HFmrEF and eGFR < 30 mL/min (AUC = 0.576; 95% CI 0.457–0.695; *p* = 0.210) (Figure 3). The diagnostic value of NT-proBNP was significantly higher in patients with eGFR ≥ 60 mL/min compared to patients with eGFR ≥ 30–<60 mL/min (*p* value for AUC comparison = 0.012) and eGFR < 30 mL/min (*p* value for AUC comparison = 0.001). The diagnostic value of NT-proBNP did not differ significantly among patients with eGFR ≥ 30–<60 mL/min and eGFR < 30 mL/min (*p* value for AUC comparison = 0.076).

### 3.4. Prognostic Performance of NT-proBNP Levels in Patients with HFmrEF and ADHF

**Figure 4:**
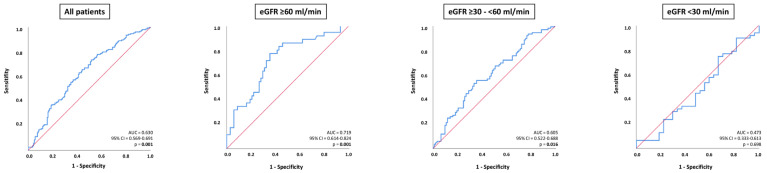
Receiver operator characteristic (ROC) curve analyses for the discrimination of 30-months all-cause mortality in patients with ADHF, as well as stratified by patients with eGFR ≥ 60 mL/min, eGFR ≥ 30–<60, and eGFR.

In all patients with ADHF, NT-proBNP was able to distinguish 30-month all-cause mortality (AUC = 0.630; 95% CI 0.569–0.691; *p* = 0.001), which was specifically evident in patients with eGFR ≥ 60 mL/min (AUC = 0.719; 95% CI 0.614–0.824; *p* = 0.001); whereas, NT-proBNP was associated with lower prognostic value in patients with eGFR ≥ 30–<60 mL/min (AUC = 0.605; 95% CI 0.522–0.688; *p* = 0.016) (Figure 4). No prognostic value of NT-proBNP levels was demonstrated for patients with eGFR < 30 mL/min (AUC = 0.473; 95% CI 0.333–0.613; *p* = 0.698). The prognostic accuracy of NT-proBNP did not significantly differ in patients with eGFR ≥ 60 mL/min compared to patients with eGFR ≥ 30–<60 mL/min (*p* value for AUC comparison = 0.092) but was statistically significantly higher in patients with eGFR ≥ 60 mL/min compared to eGFR < 30 mL/min (*p* value for AUC comparison = 0.001). There was no statistically significant difference regarding the diagnostic value of NT-proBNP in patients with eGFR ≥ 30–<60 mL/min and eGFR < 30 mL/min (*p* value for AUC comparison = 0.344).

**Figure 5:**
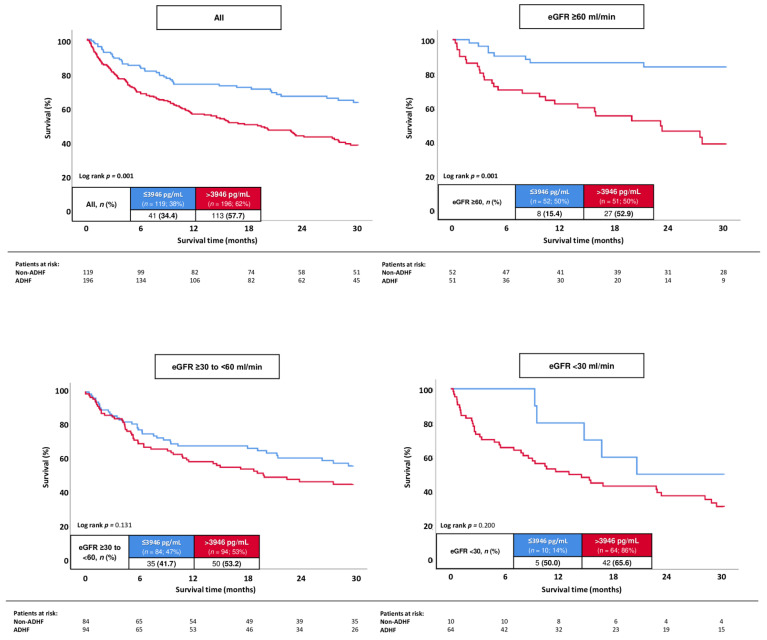
Kaplan–Meier analyses investigating the prognostic value of NT-proBNP levels regarding the risk of all-cause mortality at 30 months in all patients with ADHF, as well as stratified by patients with eGFR ≥ 60 mL/min, eGFR ≥ 30–<60, and eGFR.

In all patients with ADHF, the best cut-off of NT-proBNP to determine 30-months all-cause mortality in HFmrEF was 3946 pg/mL, with a corresponding sensitivity of 73.4% and specificity of 48.4%, respectively. Patients with higher NT-proBNP levels were associated with a statistically significant higher risk of 30-months all-cause mortality compared to patients with lower values (57.7% vs. 34.4%; log rank *p* = 0.001; HR = 2.036; 95% CI 1.423–2.912; *p* = 0.001) (Figure 5). This association was observed in the sub-group of patients with ADHF and eGFR ≥ 60 mL/min (52.9% vs. 15.4%; log rank *p* = 0.001; HR = 4.622; 95% CI 2.091–10.217; *p* = 0.001), whereas no association of NT-proBNP levels with the risk of all-cause mortality at 30 months was observed in patients with ADHF and eGFR ≥ 30–<60 (53.2% vs. 41.7%; log rank *p* = 0.131; HR = 1.716; 95% CI 0.822–1.916; *p* = 0.132) and eGFR < 30 mL/min (65.6% vs. 50.0%; log rank *p* = 0.200; HR = 1.818; 95% CI 0.718–4.600; *p* = 0.207).

Even after multivariable adjustment, patients with NT-proBNP levels > 3946 pg/mL were associated with a significant higher risk of 30-months all-cause mortality compared to patients with lower values (HR = 1.712; 95% CI 1.166–2.512; *p* = 0.006) (Table 4). In line with this, increasing age (HR = 1.032; 95% CI 1.011–1.052; *p* = 0.002) and the presence of prior congestive HF (HR = 1.450; 95% CI 1.034–2.034; *p* = 0.031) were associated with an increased risk of 30-months all-cause mortality. The prognostic value of NT-proBNP was specifically evident after multivariable adjustment in the sub-group of patients with GFR ≥ 60 mL/min (HR = 4.841; 95% CI 2.149–10.907; *p* = 0.001).

## 4. Discussion

The present study aimed to investigate the diagnostic and prognostic value of NT-proBNP levels in a large retrospective cohort of consecutive patients hospitalized with HFmrEF. The main findings of this study can be summarized as follows:

- NT-proBNP levels were higher in patients with ADHF as compared to patients without within the entire study cohort, as well as in patients with eGFR ≥ 30 mL/min. NT-proBNP levels did not differ in patients with ADHF vs. without ADHF and eGFR < 30 mL/min;
- In line with this, NT-proBNP levels discriminated the presence of ADHF within the entire study cohort (AUC = 0.777); whereas, the diagnostic value of NT-proBNP was lower in patients with impaired renal function;
- Furthermore, NT-proBNP levels predicted the risk of 30-months all-cause mortality in patients with HFmrEF and ADHF, especially in patients with preserved renal function and eGFR ≥ 60 mL/min. The prognostic impact of NT-proBNP was confirmed, even after multivariable adjustment.

In addition to clinical signs of congestion and patients’ symptoms, the measurement of blood-derived biomarkers, especially the measurement of natriuretic peptides, such as BNP and NT-proBNP, has been embedded into daily clinical practice for the diagnostic decision making and treatment of HF. From a pathophysiological point of view, BNP causes diuresis and natriuresis and further leads to smooth muscle relaxation; whereas, NT-proBNP is physiologically inactive [31]. By this point, the high diagnostic value of NT-proBNP with regard to the presence of ADHF was yet demonstrated both in patients with HFrEF and HFpEF, related to increased production and elevated plasma concentrations in patients with HF. For instance, Ibrahim et al. suggested NT-proBNP was useful in identifying patients with ADHF with an AUC of 0.926 in Asia and 0.866 in the Western world, including 1106 patients admitted to an emergency department with breathlessness [32]. Of note, the diagnosis of ADHF may be improved when incorporating baseline characteristics, clinical characteristics, and the measurement of eGFR and hemoglobin in addition to the measurement of NT-proBNP—this approach, the so-called CoDE-HF decision support tool, was recently introduced, including 10,369 patients with suspected ADHF, and revealed an AUC of 0.846 in patients with previous HF and of 0.925 in patients without prior HF, respectively [14]. The present study confirms the high diagnostic accuracy of NT-proBNP measurement for the diagnosis of ADHF in patients with HFmrEF, which was specifically observed in patients with preserved renal function. Other than the diagnostic value of NT-proBNP levels, many studies suggested higher NT-proBNP levels being associated with impaired prognosis among patients with HF [12,17,20,32,33] and atrial fibrillation [34,35]; whereas, heterogeneous findings concerning their prognostic impact in septic and cardiogenic shock was demonstrated [36,37,38]. In line with this, Kang et al. demonstrated that patients with higher NT-proBNP levels had an increased risk of all-cause mortality and rehospitalization for worsening HF after 1 year, irrespective of the presence of HFrEF and HFpEF, including 1670 patients enrolled in the Korean Heart Failure registry [39]. These findings were confirmed by Salah et al., suggesting a comparable prediction of all-cause mortality in HFrEF and HfpEF; whereas, specifically, a higher burden of comorbidities contributed to the prognosis of patients with HFpEF than HFrEF [33]. This is of major importance since the number of comorbidities in individuals with HF is steadily increasing.

From this perspective, especially, the number of patients with HF and concomitant arterial hypertension, atrial fibrillation, and chronic kidney disease was shown to increase from 2001 to 2016 [5]. In line with this, even 35% of patients with HFmrEF included in the present study suffered from concomitant chronic kidney disease. Thus, the diagnostic and prognostic value of NT-proBNP measurement was shown to be limited in patients with impaired renal function, especially in patients with eGFR < 30 mL/min. The current literature is characterized by heterogeneous findings concerning the prognostic value of NT-proBNP levels in patients with impaired renal function. For instance, Horii et al. suggested NT-proBNP was associated with reliable discrimination of all-cause mortality irrespective of renal function; whereas, NT-proBNP was associated with better discrimination of all-cause mortality compared to BNP in patients with chronic kidney disease stages 4–5 [40]. In line, NT-proBNP levels predicted the risk of mortality among 341 patients with congestive HF, irrespective of the presence or absence of chronic kidney disease [41]. In contrast, lower prognostic accuracy with regard to all-cause mortality was observed in patients with chronic kidney disease stage 3b (AUC = 0.616) as compared to patients with stage 3a (AUC = 0.697), including 168 patients of at least 80 years of age [42]. Poor prediction of all-cause mortality in patients with advanced stages of chronic kidney disease may be attributed to the very high risk of all-cause mortality related to chronic kidney disease itself (i.e., at least 50% within the present study in patients with eGFR < 30 mL/min at 30 months). Thus, the presence of chronic kidney disease was recently shown to increase the risk of both ventricular tachyarrhythmias and sudden cardiac death [43,44]. Further studies are, therefore, necessary to investigate the prognostic role of NT-proBNP in patients with advanced stages of chronic kidney disease with regard to the risk of HF-related mortality and to separate its impact on cardiovascular death.

Within the present study, NT-proBNP levels were associated with prognosis despite a high rate of patients with an optimal pharmacological treatment, including beta-blockers and inhibitors of the renin–angiotensin–aldosterone system. However, within the present study, specifically, the proportion of patients treated with a sodium-glucose-linked transporter 2 (SGLT2) inhibitor was rather low and only 6.7% of patients without ADHF and 4.7% with ADHF were treated with a SGLT2 inhibitor. The low prescription rates of SGLT2 inhibitors are in line with a previous study from the Swedish HF registry; whereas, only 5.5% of patients with concomitant diabetes mellitus were treated with a SGLT2 inhibitor from 2016 to 2018 [45]. This may be in accordance with the upgrade within the updated ESC HF guidelines; whereas, specifically, treatment with SGLT2 inhibitors gained more importance in 2023 [46]. From this perspective, the prognostic impact of SGLT2 inhibitors may be superior in patients with increased NT-proBNP levels and may decrease NT-proBNP levels in HF patients [47]. However, further studies are needed in patients with HFmrEF receiving an optimal HF pharmacotherapy, including treatment with SGLT2 inhibitors after being embedded into daily clinical practice.

The findings of the present study, suggesting the superior diagnostic and prognostic value of NT-proBNP in patients with HFmrEF and preserved renal function, are of the utmost importance given ongoing demographic changes and the aging of the population. In line with this, the burden of cardiovascular and non-cardiovascular comorbidities was demonstrated to increase, leading to a higher proportion of patients with chronic kidney disease [5,48]. Specifically in patients with advanced stages of chronic kidney disease, both the diagnostic and prognostic accuracy of NT-proBNP were limited within the present all-comer study, including patients hospitalized with HFmrEF. Furthermore, the limited diagnostic capacity of NT-proBNP was not yet demonstrated in patients with obesity and atrial fibrillation [49]. Given these findings, further studies are warranted to improve risk stratification for patients with HF and chronic kidney disease. From this perspective, the combined assessment of biomarkers may improve risk stratification. The measurement of soluble (s)ST2 was recently shown to improve the prognostic accuracy when combined with NT-proBNP measurement [50]. Especially in patients with chronic kidney disease, sST2 was shown to be associated with the risk of developing HF, including 3314 patients [51]. Therefore, further studies are needed to evaluate the diagnostic and prognostic accuracy of sST2 in patients with HF and chronic kidney disease.

## 5. Study Limitations

This study has several limitations. Due to the retrospective and single-center study design, results may be influenced by measured and unmeasured confounding variables. NT-proBNP was not measured in a large proportion of patients, which may bias the conclusions diagnostically, especially for the non-ADHF group. In line with this, serial measurements of NT-proBNP levels were only available in a minor part of the study population and, therefore, not included. No information on eGFR beyond index hospitalization was available. The proportion of patients with eGFR < 30 mL/min was rather low within the present study. In addition, changes in LVEF during the course of follow-up were available in a minor part of the study population and, therefore, were beyond the scope of the present study. Patients with ambulatory visits only were not included in the present study. This may further impact the findings of the present study by presumably including a higher proportion of sicker patients. Furthermore, no sub-analyses were performed further stratifying by the patients’ age, sex, body mass index, or atrial fibrillation, which may further affect NT-proBNP levels. Finally, causes of death beyond index hospitalization were not available for the present study.

## 6. Conclusions

NT-proBNP was associated with reliable diagnostic and prognostic value and independently predicted the risk of 30-months all-cause mortality, especially in patients with preserved renal function. In contrast, NT-proBNP revealed neither diagnostic nor prognostic value in patients with eGFR < 30 mL/min.

## Figures and Tables

**Table 1:** Baseline characteristics.

	Non-ADHF (*n* = 440)		ADHF (*n* = 315)		*p* Value
**Age**, median (IQR)	70	(60–79)	80	(73–85)	**0.001**
**Male sex**, *n* (%)	304	(69.1)	181	(57.5)	**0.001**
**Body mass index**, kg/m^2^, median (IQR)	26.9	(23.9–30.9)	26.3	(23.7–31.1)	0.602
**SBP**, mmHg, median (IQR)	140	(120–157)	140	(120–160)	0.773
**DBP**, mmHg, median (IQR)	78	(68–89)	75	(64–89)	0.097
**Heart rate**, bpm, median (IQR)	80	(68–96)	83	(70–99)	0.089
**Medical history**, *n* (%)					
Coronary artery disease	171	(38.9)	130	(41.3)	0.506
Prior myocardial infarction	100	(22.7)	76	(24.1)	0.654
Prior PCI	124	(28.2)	98	(31.1)	0.384
Prior CABG	42	(9.5)	34	(10.8)	0.574
Prior valvular surgery	23	(5.2)	16	(5.1)	0.928
Congestive heart failure	135	(30.7)	155	(49.2)	**0.001**
Decompensated heart failure < 12 months	44	(10.0)	63	(20.0)	**0.001**
Prior ICD	14	(3.2)	3	(1.0)	**0.042**
Prior sICD	4	(0.9)	1	(0.3)	0.323
Prior CRT-D	6	(1.4)	4	(1.3)	0.911
Prior Pacemaker	23	(5.2)	38	(12.1)	**0.001**
Chronic kidney disease	97	(22.0)	168	(53.3)	**0.001**
Peripheral artery disease	41	(9.3)	43	(13.7)	0.062
Stroke	49	(11.1)	49	(15.6)	0.075
Liver cirrhosis	7	(1.6)	9	(2.9)	0.234
Malignancy	68	(15.5)	45	(14.3)	0.657
COPD	53	(12.0)	54	(17.1)	**0.048**
**Cardiovascular risk factors**, *n* (%)					
Arterial hypertension	316	(71.8)	269	(85.4)	**0.001**
Diabetes mellitus	136	(30.9)	155	(49.2)	**0.001**
Hyperlipidemia	143	(32.5)	102	(32.4)	0.973
Smoking					
Current	100	(22.7)	32	(10.2)	**0.001**
Former	94	(21.4)	65	(20.6)	0.809
Family history	57	(13.0)	21	(6.7)	**0.005**
**Department of index admission**, *n* (%)					
Internal medicine	387	(88.0)	271	(86.0)	0.082
Surgery	19	(4.3)	17	(5.4)	
Neurology	11	(2.5)	5	(1.6)	
Orthopedics	3	(0.7)	6	(1.9)	
Urology	8	(1.8)	10	(3.2)	
Others	12	(2.7)	6	(1.9)	
**Comorbidities at index hospitalization**, *n* (%)					
Acute coronary syndrome					
Unstable angina	25	(5.7)	7	(2.2)	**0.020**
STEMI	70	(15.9)	14	(4.4)	**0.001**
NSTEMI	74	(16.8)	35	(11.1)	**0.028**
Cardiogenic shock	16	(3.6)	17	(5.4)	0.243
Atrial fibrillation	148	(33.6)	185	(58.7)	**0.001**
Cardiopulmonary resuscitation	15	(3.4)	11	(3.5)	0.951
Out-of-hospital	8	(1.8)	3	(1.0)	0.328
In-hospital	7	(1.6)	8	(2.5)	0.357
Stroke	21	(4.8)	7	(2.2)	0.067
**Medication on admission**, *n* (%)					
ACE-inhibitor	142	(32.3)	125	(39.7)	**0.036**
ARB	101	(23.0)	80	(25.4)	0.438
Beta-blocker	221	(50.2)	221	(70.2)	**0.001**
Aldosterone antagonist	41	(9.3)	43	(13.7)	0.062
ARNI	5	(1.1)	3	(1.0)	0.808
SGLT2-inhibitor	12	(2.7)	8	(2.5)	0.874
Loop diuretics	123	(28.0)	196	(62.2)	**0.001**
Statin	201	(45.7)	153	(48.6)	0.433
ASA	149	(33.9)	97	(30.8)	0.375
P2Y12-inhibitor	39	(8.9)	39	(12.4)	0.117
DOAC	84	(19.1)	109	(34.6)	**0.001**
Vitamin K antagonist	22	(5.0)	29	(9.2)	**0.023**

ACE, angiotensin-converting-enzyme; ARB, angiotensin receptor blocker; ARNI, angiotensin receptor neprilysin inhibitor; ASA, acetylsalicylic acid; CABG, coronary artery bypass grafting; COPD, chronic obstructive pulmonary disease; CRT-D, cardiac resynchronization therapy with defibrillator; DBP, diastolic blood pressure; DOAC, directly acting oral anticoagulant; IQR, interquartile range; (N)STEMI, non-ST-segment elevation myocardial infarction; SBP, systolic blood pressure; SGLT2, sodium glucose linked transporter 2; (s) ICD, (subcutaneous) implantable cardioverter defibrillator. Level of significance *p* ≤ 0.05. Bold type indicates statistical significance.

**Table 2:** Heart-failure-related and procedural data.

	Non-ADHF (*n* = 440)		ADHF (*n* = 315)		*p* Value
**Heart failure etiology**, *n* (%)					
Ischemic	287	(65.2)	178	(56.5)	**0.001**
Non-ischemic cardiomyopathy	37	(8.4)	21	(6.7)	
Hypertensive cardiomyopathy	21	(4.8)	21	(6.7)	
Congenital heart disease	1	(0.2)	0	(0.0)	
Valvular heart disease	11	(2.5)	28	(8.9)	
Tachycardia associated	14	(3.2)	21	(6.7)	
Tachymyopathy	10	(2.3)	9	(2.9)	
Pacemaker-induced cardiomyopathy	2	(0.5)	4	(1.3)	
Unknown	57	(13.0)	33	(10.5)	
**NYHA functional class**, *n* (%)					
I/II	334	(75.9)	66	(20.9)	**0.001**
III	85	(19.3)	141	(44.8)	
IV	21	(4.8)	108	(34.3)	
**Echocardiographic data**					
LVEF, %, median (IQR)	45 (45–47)		45 (44–47)		0.300
IVSd, median (IQR)	12 (10–13)		12 (11–13)		**0.008**
LVEDD, mm, median (IQR)	49 (45–54)		49 (45–54)		0.384
TAPSE, mm, median (IQR)	20 (17–23)		20 (16–22)		**0.012**
LA diameter, mm, median (IQR)	40 (36–46)		45 (40–49)		**0.001**
LA surface, cm^2^, median (IQR)	20 (17–24)		24 (21–29)		**0.001**
E/A, median (IQR)	0.9 (0.7–1.3)		1.0 (0.6–1.5)		0.254
E/E`, median (IQR)	9.5 (6.5–13.5)		13.8 (7.4–19.6)		**0.001**
Diastolic dysfunction, *n* (%)	290	(65.9)	230	(73.0)	**0.038**
Moderate-severe aortic stenosis, *n* (%)	26	(5.9)	42	(13.3)	**0.001**
Moderate-severe aortic regurgitation, *n* (%)	15	(3.4)	28	(8.9)	**0.001**
Moderate-severe mitral regurgitation, *n* (%)	36	(8.2)	86	(27.3)	**0.001**
Moderate-severe tricuspid regurgitation, *n* (%)	49	(11.1)	105	(33.3)	**0.001**
VCI, mm, median (IQR)	17 (13–22)		23 (17–27)		**0.001**
Aortic root, mm, median (IQR)	33 (30–36)		33 (29–36)		0.064
**Coronary angiography**, *n* (%)	248	(56.4)	126	(40.0)	**0.001**
No evidence of coronary artery disease	42	(16.9)	30	(23.8)	**0.011**
1-vessel disease	52	(21.0)	24	(19.0)	
2-vessel disease	53	(21.4)	11	(8.7)	
3-vessel disease	101	(40.7)	61	(48.4)	
CABG	21	(8.5)	10	(7.9)	0.860
Chronic total occlusion	27	(10.9)	13	(10.3)	0.866
PCI, *n* (%)	151	(60.9)	58	(46.0)	**0.006**
Sent to CABG, *n* (%)	9	(3.6)	7	(5.6)	0.384
**Baseline laboratory values**, median (IQR)					
Potassium, mmol/L	3.9 (3.6–4.2)		3.8 (3.5–4.2)		**0.015**
Sodium, mmol/L	139 (137–141)		139 (137–141)		0.567
Creatinine, mg/dL	1.01 (0.85–1.30)		1.35 (1.01–1.98)		**0.001**
eGFR, mL/min/1.73 m^2^	72 (53–91)		49 (32–69)		**0.001**
Hemoglobin, g/dL	12.8 (10.9–14.3)		11.2 (9.3–12.7)		**0.001**
WBC count, ×10^9^/L	8.20 (6.57–10.21)		8.38 (6.52–10.59)		0.540
Platelet count, ×10^9^/L	224 (176–287)		237 (175–299)		0.260
HbA1c, %	5.8 (5.4–6.5)		6.2 (5.6–7.3)		**0.004**
LDL-cholesterol, mg/dL	96 (74–132)		87 (63–119)		**0.007**
HDL-cholesterol, mg/dL	42 (34–52)		42 (33–51)		0.334
C-reactive protein, mg/L	14 (3–45)		21 (8–59)		**0.001**
NT-pro BNP, pg/mL	1655 (481–3326)		5394 (2510–11,883)		**0.001**
Cardiac troponin I, µg/L	0.05 (0.02–0.42)		0.04 (0.02–0.15)		0.096
**Medication at discharge**, *n* (%)					
ACE-inhibitor	236	(54.8)	141	(47.8)	0.065
ARB	113	(26.2)	85	(28.8)	0.441
Beta-blocker	349	(81.0)	254	(86.1)	0.070
Aldosterone antagonist	62	(14.4)	72	(24.4)	**0.001**
ARNI	7	(1.6)	4	(1.4)	0.771
SGLT2-inhibitor	29	(6.7)	14	(4.7)	0.266
Loop diuretics	169	(39.2)	267	(90.5)	**0.001**
Statin	309	(71.7)	191	(64.7)	**0.047**
Digitalis	16	(3.7)	17	(5.8)	0.193
Amiodarone	14	(3.2)	9	(3.1)	0.881
ASA	238	(55.2)	119	(40.3)	**0.001**
P2Y12-inhibitor	185	(42.9)	86	(29.2)	**0.001**
DOAC	132	(30.6)	136	(46.1)	**0.001**
Vitamin k antagonist	23	(5.3)	26	(8.8)	0.067

ACE, angiotensin-converting enzyme; ADHF, acute decompensated heart failure; ARB, angiotensin receptor blocker; ARNI, angiotensin receptor neprilysin inhibitor; ASA, acetylsalicylic acid; CABG, coronary artery bypass grafting; DOAC, directly acting oral anticoagulant; eGFR, estimated glomerular filtration rate; HbA1c, glycated hemoglobin; HDL, high-density lipoprotein; IQR, interquartile range; IVSd, Interventricular septal end diastole; LA, left atrial; LDL, low-density lipoprotein; LVEDD, Left ventricular end-diastolic diameter; LVEF, left ventricular ejection fraction; NT-pro BNP, aminoterminal pro-B-type natriuretic peptide; NYHA, New York Heart Association; PCI, percutaneous coronary intervention; TAPSE, tricuspid annular plane systolic excursion; VCI, Vena cava inferior; WBC, white blood cell. Level of significance *p* ≤ 0.05. Bold type indicates statistical significance.

**Table 3:** Correlations of NT-proBNP with clinical, echocardiographic, and laboratory data.

	r	*p* Value
Age	0.382	**0.001**
Body mass index (kg/m^2^)	−0.202	**0.001**
LVEF (%)	−0.098	**0.007**
TAPSE (mm)	−0.089	**0.015**
eGFR (mL/min/1.73 m^2^)	−0.476	**0.001**
Hemoglobin (g/dL)	−0.466	**0.001**
Cardiac troponin I (µg/L)	0.241	**0.001**
LDL-cholesterol (mg/dL)	−0.211	**0.001**
HDL-cholesterol (mg/dL)	−0.069	0.154
HbA1c (%)	0.087	0.090
C-reactive protein (mg/L)	0.319	**0.001**

eGFR, estimated glomerular filtration rate; HbA1c, glycated hemoglobin; HDL, high-density lipoprotein; LDL, low-density lipoprotein; LVEF, left ventricular ejection fraction; NT-pro BNP, aminoterminal pro-B-type natriuretic peptide; TAPSE, tricuspid annular plane systolic excursion. Level of significance *p* < 0.05. Bold type indicates statistical significance.

**Table 4:** Multivariable Cox regression analyses with regard to 30-month all-cause mortality in patients with ADHF and HFmrEF.

Variables	HR	95% CI	*p* Value
**Model 1**			
Age (per year increase)	1.032	1.011–1.052	**0.002**
Males	1.141	0.816–1.595	0.442
Prior congestive heart failure	1.450	1.034–2.034	**0.031**
Atrial fibrillation	1.076	0.756–1.531	0.684
Ischemic cardiomyopathy	0.858	0.618–1.191	0.359
eGFR (per mL/min increase)	0.996	0.990–1.003	0.288
Hemoglobin (per g/dL increase)	0.927	0.856–1.003	0.061
NT-proBNP > 3946 pg/mL	1.712	1.166–2.512	**0.006**
Model 2 *			
eGFR ≥ 60 mL/min	4.841	2.149–10.907	**0.001**
eGFR ≥ 30–<60 mL/min	1.225	0.769–1.952	0.392
eGFR < 30 mL/min	2.106	0.773–5.741	0.145

CI, confidence interval; eGFR, estimated glomerular filtration rate; HR, hazard ratio; NT-pro BNP, aminoterminal pro-B-type natriuretic peptide. Level of significance *p* < 0.05. Bold type indicates statistical significance. * Model 2: multivariable Cox regression analyses were additionally performed and stratified by eGFR and HR; additionally, 95% CI and *p*-values were provided for NT-proBNP > 3946 pg/mL.

## Data Availability

The datasets used and/or analyzed during the current study are available from the corresponding author upon reasonable request.
